# Different Effects of Maternal Low-Isoflavone Soy Protein and Genistein Consumption on Hepatic Lipid Metabolism of 21-Day-Old Male Rat Offspring

**DOI:** 10.3390/nu9091039

**Published:** 2017-09-20

**Authors:** Anna Han, Sae Bom Won, Young Hye Kwon

**Affiliations:** 1 Department of Food and Nutrition, Seoul National University, Seoul 08826, Korea; anteanna@naver.com (A.H.); newspring@snu.ac.kr (S.B.W.); 2 Research Institute of Human Ecology, Seoul National University, Seoul 08826, Korea

**Keywords:** amino acid, lipid metabolism, maternal diet, offspring liver, PPARα, soy protein isolate

## Abstract

Amino acid composition and isoflavone are alleged contributors to the beneficial effects of soy protein isolate (SPI) on lipid metabolism. Therefore, we investigated the contributing component(s) of SPI in a maternal diet to the regulation of lipid metabolism in offspring. We also determined serum parameters in dams to investigate specific maternal cues that might be responsible for this regulation. Female rats were fed either a casein (CAS), a low-isoflavone SPI, or a casein plus genistein (GEN, 250 mg/kg) diet for two weeks before mating, as well as during pregnancy and lactation. Male offspring (CAS, SPI and GEN groups) were studied 21 days after birth. The SPI group had lower serum triglyceride levels than the other groups. Serum cholesterol was reduced in both the SPI and GEN groups compared with the CAS group. Expressions of target genes of peroxisome proliferator-activated receptor α were altered in the SPI group. Serum aromatic amino acid levels in dams were associated with serum triglyceride in offspring. In conclusion, the maternal consumption of a low-isoflavone SPI diet or a casein diet containing genistein has different effects on the lipid metabolism of their offspring; however, more profound effects were observed in the SPI group. Therefore, the altered lipid metabolism of offspring may be attributed to amino acid composition in maternal dietary protein sources.

## 1. Introduction

Several studies in humans and experimental animals have demonstrated that the early life environments may alter overall homeostatic regulatory mechanisms, thereby playing a critical role in influencing the susceptibility of offspring to the later development of certain diseases, as well as fetal programming [1]. Especially, nutrition during fetal and neonatal periods is one of the major environmental factors that affect the risk of chronic diseases such as obesity, hypertension, cardiovascular diseases, and diabetes in adulthood [2,3].

It has been shown that the consumption of a soy protein diet reduces serum triglyceride and cholesterol in rats [4,5]. In particular, soy isoflavone has been shown to alleviate metabolic diseases by reducing serum and hepatic lipid levels [6]. Similarly, exposure to dietary soy protein isolate (SPI) with isoflavone throughout the in utero, neonatal, and adult periods reduced hepatosteatosis compared with casein [7]. Although the maternal consumption of genistein or daidzein during pregnancy and lactation was reported to reduce the width to length ratio of myocytes, which may be related to the cardioprotective effects on their offspring during adulthood [8], most previous studies have investigated only the effect of supplementary genistein in a casein-based diet. 

Conversely, another study showed that body weight and fat pad weight between the offspring of dams placed on a soy protein diet (with isoflavone) and the offspring of dams placed on a casein plus genistein diet were different in spite of similar serum genistein levels in dams [9]. Similarly, dietary soy protein with low isoflavone reduced plasma and hepatic cholesterol and/or triglyceride in rats [10,11]. These results suggest that other SPI-derived dietary components may also play important roles in ameliorating metabolic diseases. Indeed, soy protein has a different amino acid composition than casein, with higher amounts of several amino acids, including cysteine, arginine, and glycine, and lower amounts of methionine. It is reported that the effect of dietary soy protein on lipid metabolism may be mediated by its low proportion of methionine among the total sulfur-containing amino acids [12]. Glycine has also been shown to stimulate insulin secretion [13], suggesting that the type of dietary protein consumed may play an important role in preventing the development of metabolic syndrome. In addition, previous studies reported the hypocholesterolemic effects of soy bioactive peptides and protein fractions [14].

Although several studies have reported that maternal diet affects metabolic disease development in adulthood, few studies have determined the alteration in organs and disease-related metabolism of young offspring. Previously, we have reported that the maternal consumption of low-isoflavone SPI significantly altered the hepatic gene expression profile of offspring. Much less effect was observed in response to genistein supplementation in the maternal diet [15]. Therefore, the aim of this study was to compare the effects of the early-life consumption of a low-isoflavone SPI diet and a casein diet supplemented with genistein on hepatic glucose and lipid metabolism of male offspring rats at postnatal day (PND) 21. 

## 2. Materials and Methods 

### 2.1. Animals and Diets

Seven-week-old virgin female Sprague-Dawley rats were obtained from the local animal facility (Orient Bio Inc., Seongnam, Korea) and were maintained in a temperature (22 ± 3 °C) and humidity (50 ± 10%)-controlled room with a 12 h dark/light cycle. The experimental procedures used in the present study were approved by Seoul National University Institutional Animal Care and Use Committee (#SNU-081006-4). As previously described [15], virgin female rats were randomly assigned into three groups after a one week acclimation period. Each group was offered the experimental diets containing casein (CAS diet), low-isoflavone SPI (SPI diet), or casein plus genistein (GEN diet: 250 mg/kg diet), which were prepared on the American Institute of Nutrition-93G formula, except that soybean oil was replaced with corn oil. The composition of diet is shown in Table 1. After two weeks of feeding, females were allowed to mate with mature males of the same strain (2:1). Female rats were maintained on their corresponding diet throughout pregnancy and lactation. Diets and water were provided *ad libitum*. The litter size and pup body weight were recorded at birth, and litters were adjusted to two females and six males to normalize the growth. Dams (CAS-D, SPI-D, and GEN-D groups) and their male offspring at age of PND 21 (CAS, SPI, and GEN groups) were sacrificed after an overnight fast. Blood samples were rapidly obtained by cardiac puncture. Tissues were removed, snap-frozen immediately in liquid nitrogen, and stored at −80 °C until use.

### 2.2. Serum Biochemical Analyses

Blood was centrifuged at 10,000× *g* for 15 min and stored at −80 °C until analyzed. Serum glucose, triglyceride, total cholesterol, and high-density lipoprotein (HDL) cholesterol levels were determined using commercial kits (Asan Pharmaceutical Co., Seoul, Korea). Serum free fatty acid levels were measured using a commercially available kit (Shinyang Diagnostics, Seoul, Korea). Serum hormones, including insulin (Millipore, Temecula, CA, USA), adiponectin (R&D Systems, Minneapolis, MN, USA), and triiodothyronine (T3; GenWay Biotech, San Diego, CA, USA), were measured using an ELISA kit. The insulin resistance index was estimated by the homeostasis model assessment of insulin resistance (HOMA-IR) with the following formula: serum glucose (mmol/L) × serum insulin (mU/L)/22.5. Serum total homocysteine levels were determined by high performance liquid chromatography (HPLC) method according to previously described protocol [16].

### 2.3. Serum Free Amino Acid Analysis

Serum free amino acid levels were determined using the HPLC method, as previously described [17]. Briefly, serum samples were mixed with 2 mmol/L norvaline as an internal standard and 20% sulphosalicylic acid to precipitate protein. After centrifugation at 12,000× *g* for 5 min at 4 °C, the supernatant containing free amino acid was removed and filtered. Samples and free amino acid standard (Agilent Technologies, Santa Clara, CA, USA) were analyzed by the Agilent 1200 HPLC equipped with an Inno C18 column (4.6 mm × 150 mm, 5 μm, Young Jin Biochrom Co., Seongnam, Korea), and the fluorescence detector. The derivatization reagents of amino acids were o-phthalaldehyde (OPA) for the primary amino acids and 9-flurorenylmethyl chloroformate (FMOC) for proline. The analytes were eluted with a gradient of eluent A (20 mm phosphate buffer, pH 7.8) and eluent B (acetonitrile:methanol:water, 45:45:10, *v*/*v*/*v*) at a flow rate of 1.5 mL/min. The fluorescence detector was set at excitation 340 nm and emission 450 nm for OPA, and excitation 266 nm and emission 305 nm for FMOC.

### 2.4. Oral Glucose Tolerance Test

At 18–19 days of age, randomly selected four males per group with each offspring obtained from a different litter underwent the oral glucose tolerance test. Overnight-fasted offspring were given a single dose of oral glucose (2 g/kg body weight), and blood samples were obtained from the tail vein periodically over a 2-h period prior to (time 0) and 30, 60, 90, and 120 min after the glucose load. Blood glucose levels were determined by using the Accu-Chek Advantage System (Roche Diagnostics, Indianapolis, IN, USA). Total area under the curve (AUC) during the oral glucose tolerance test was calculated using the trapezoidal rule.

### 2.5. Hepatic Lipid Analyses

Total lipids were extracted according to the method of Folch et al. [18]. Briefly, hepatic tissue was homogenized in 20 volumes (*w*/*v*) of ice-cold phosphate buffered saline, and the protein content was measured using the commercial kit (Bio-Rad, Hercules, CA, USA). The homogenates containing 300 μg of protein (1 mg/mL) were incubated in 1.2 mL of methanol-chloroform (1:2, *v*/*v*) at 4 °C for 3 h. After incubation, 240 μL of 0.88% KCl was added for the aggregation of non-lipid contents, and centrifuged at 1000× *g* for 15 min at 4 °C. The bottom layer was transferred to the new tube, and hepatic triglyceride and cholesterol levels were determined by enzymatic colorimetric methods using commercial kits (Asan Pharmaceutical Co., Seoul, Korea).

### 2.6. Total RNA Extraction, Microarray Analysis and Real-Time PCR

Total RNA of liver tissue was isolated using RNAiso Plus (Takara Bio Inc., Shiga, Japan), and the amount of RNA was measured using Quant-iT™ RNA Assay Kit (Invitrogen, Carlsbad, CA, USA). RNA purity and integrity were analyzed on an Agilent 2100 Bioanalyzer (Agilent Technologies, Santa Clara, CA, USA). Microarray hybridization was performed with the Illumina RatRef-12 v1.0 Expression BeadChip platform (San Diego, CA, USA). Samples from each group (*n* = 4) were analyzed by microarray. A detailed description of microarray hybridization and analysis was given in a previous study [15]. Further comprehensive analysis was performed on 119 differentially expressed genes with the fold change ≥1.5 between two groups (*p* < 0.05). For real-time PCR, cDNA was synthesized using 2 μg of total RNA with the Superscript™ II Reverse Transcriptase (Invitrogen, Carlsbad, CA, USA). Amplification reactions were performed using a StepOne™ Real-time PCR System (Applied Biosystems, Foster City, CA, USA) according to manufacturer’s protocol. The selective gene expressions were determined by TaqMan™- or SYBR*^®^* green-based detection. PCR primers are described in Supplementary Tables S1 and S2, respectively. Beta-actin was used as an endogenous control. Relative gene expression levels were analyzed using the *2*^−ΔΔCt^ method.

### 2.7. Tissue Extraction and Immunoblotting

Liver tissues were homogenized in ice-cold protein lysis buffer. Equal amounts of protein were loaded into the lanes of sodium dodecyl sulfate polyacrylamide gel electrophoresis gel, separated, and blotted onto a polyvinylidene difluoride membrane. After being blocked with 5% nonfat milk or bovine serum albumin in Tris-buffered saline, membranes were probed with anti-phosphorylated AMP-activated protein kinase alpha (p-AMPKα: Cell Signaling, Danvers, MA, USA) or beta-actin (Sigma-Aldrich, St. Louis, MO, USA). The membranes were then incubated with a secondary antibody for chemiluminescent detection. The band intensities were quantified with Quantity One software (Bio-Rad, Hercules, CA, USA).

### 2.8. Statistical Analysis

Statistical analyses were performed using IBM SPSS Statistics version 19.0 software (IBM SPSS Inc., Armonk, NY, USA). For all experiments, one-way analysis of variance (ANOVA) followed by Duncan’s multiple range test or an independent *t*-test was used to determine statistical significance among the groups. Data were expressed as means ± standard error of the mean (SEM), and differences were considered statistically significant at *p* < 0.05. Correlation between two variables was analyzed by Pearson correlation coefficient.

## 3. Results

### 3.1. Effects of Maternal Diet on Maternal Body Weight Changes and Serum Biochemical Parameters

The body weight of dams fed an SPI or a GEN diet were not significantly different from those fed a CAS diet during the whole experimental period (Supplementary Figure S1). There was no significant difference in body weights of dams on postpartum day 21 (Table 2). Serum triglyceride, total cholesterol, and HDL cholesterol levels were significantly lower in dams fed an SPI diet compared with those in dams fed a CAS diet. Only HDL cholesterol levels were significantly lower in dams fed a GEN diet compared with dams fed a CAS diet. No significant differences in serum glucose, free fatty acids, and homocysteine levels were observed among groups. Consistent with observations in serum, hepatic triglyceride and cholesterol levels were significantly lower in the SPI-D group compared with the CAS-D and GEN-D groups.

We also determined the serum levels of several hormones responsible for fetal growth and development, including insulin, adiponectin, and T3 [19,20], in dams to determine whether endocrine status was altered by diet (Table 2). The experimental diet did not affect the serum insulin or adiponectin levels of dams, but genistein supplementation significantly reduced serum T3 levels.

### 3.2. Effect of Maternal Diet on Serum Amino Acid Levels in Dams

As shown in Table 3, serum concentrations of aromatic amino acid (AAA), including tyrosine and tryptophan, were significantly higher in the SPI-D group compared with the CAS-D and GEN-D groups. Conversely, serum valine levels were significantly lower in the SPI-D group compared with the CAS-D group. The ratio of branched-chain amino acid (BCAA: isoleucine, leucine and valine) to AAA (phenylalanine, tryptophan and tyrosine) (BCAA/AAA) was significantly reduced by consumption of an SPI diet.

### 3.3. Effects of Maternal Diet on Growth and Glucose Metabolism of Offspring

**Figure 1:**
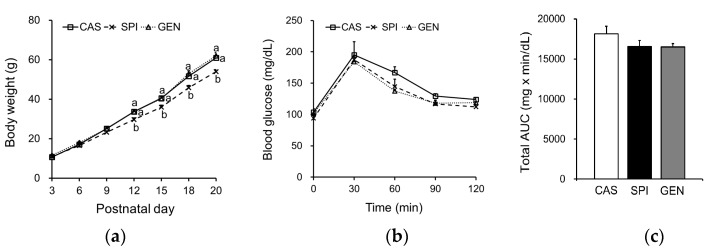
Effects of maternal diet on (**a**) body weight change (*n* = 14–17) and (**b**,**c**) glucose metabolism in male offspring (*n* = 4). Blood glucose curve and total area under the curve (AUC) in response to a glucose-loading test. Data are means ± standard error of the mean. Means with different superscripts are significantly different at *p* < 0.05. CAS, offspring of dams fed a casein diet; SPI, offspring of dams fed a low-isoflavone soy protein isolate diet; GEN, offspring of dams fed a casein plus genistein diet.

The body weights of offspring of dams fed an SPI diet were significantly lower than those of the other groups, starting from PND 12, up to the end of the experiment (Figure 1a). There were no significant differences in serum glucose levels (CAS: 65.1 ± 4.6; SPI: 59.3 ± 4.4; GEN: 55.2 ± 4.2 mg/dL), serum insulin levels (CAS: 0.74 ± 0.37; SPI: 0.46 ± 0.12; GEN: 0.72 ± 0.29 ng/mL), and HOMA-IR (CAS: 2.7 ± 1.3; SPI: 1.6 ± 0.6; GEN: 2.6 ± 1.3) among the offspring groups, although the values of the SPI group tended to be lower than those of the other two groups. We also investigated whether insulin signaling was altered by the maternal diet using the oral glucose tolerance test, and observed no significant differences in blood glucose levels and total AUC for glucose among the groups (Figure 1b,c).

### 3.4. Effects of Maternal Diet on Lipid Metabolism of Offspring

**Figure 2:**
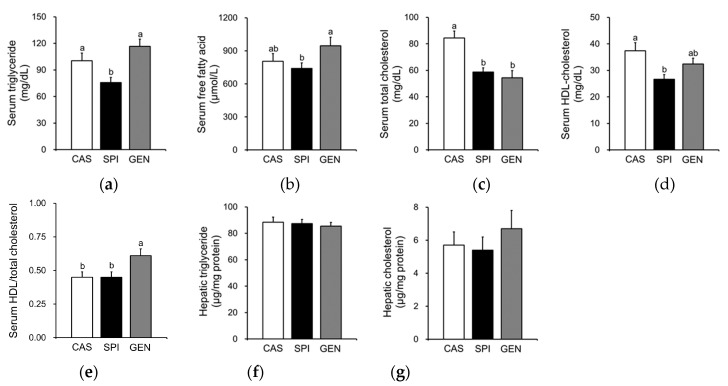
Effects of maternal diet on lipid metabolism of male offspring. (**a**) Serum triglyceride, (**b**) free fatty acid, (**c**) total cholesterol, (**d**) high-density lipoprotein (HDL) cholesterol levels, and (**e**) the ratio of HDL cholesterol to total cholesterol (*n* = 8–10); (**f**) Hepatic triglyceride and (**g**) cholesterol levels (*n* = 9–11). Data are means ± standard error of the mean. Means with different superscripts are significantly different at *p* < 0.05.

Serum triglyceride levels were significantly lower in the SPI group than the CAS and GEN groups (Figure 2a), and free fatty acid levels were significantly lower in the SPI group than the GEN group (Figure 2b). Maternal intake of SPI and GEN diets significantly reduced serum total cholesterol levels in offspring (Figure 2c). Although serum HDL cholesterol levels were significantly lower in the SPI group compared with the CAS group (Figure 2d), the ratio of HDL cholesterol to total cholesterol was not significantly different between the CAS and SPI groups (Figure 2e). In contrast, the ratio was significantly increased by maternal consumption of the GEN diet. Unlike their dams, the offspring did not show any significant differences in hepatic triglyceride or cholesterol concentrations (Figure 2f,g), which suggests that the decreases in serum triglyceride levels in the SPI group were not simply due to hepatic triglyceride accumulation.

### 3.5. Effects of Maternal Diet on Hepatic Gene Expressions Involved in Lipid Metabolism of Offspring

**Figure 3:**
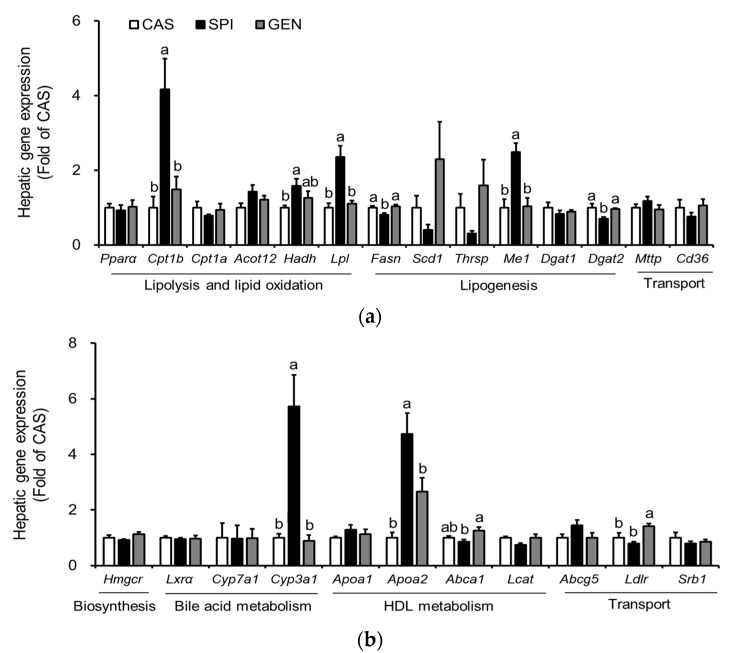
Effects of maternal diet on hepatic gene expression related to the lipid metabolism of male offspring. (**a**) Triglyceride metabolism; (**b**) Cholesterol metabolism. Relative mRNA level of each gene was determined by real-time PCR. Beta-actin was used as an endogenous control. Data are means ± standard error of the mean (*n* = 4). Means with different superscripts are significantly different at *p* < 0.05.

Previously, gene ontology analysis of microarray data showed that 58 genes related to “lipid, fatty acid and steroid metabolism” are significantly affected by maternal diet [15]. Therefore, we confirmed the mRNA levels of major genes involved in triglyceride metabolism (Figure 3a) and cholesterol metabolism (Figure 3b) by real-time PCR. A triglyceride-lowering effect of SPI was supported by higher hepatic mRNA levels of several genes involved in lipolysis and lipid oxidation, including *Lpl*, *Cpt1b* and *Hadh* compared with those of the CAS group. An increased expression of *Lpl* has been reported to be involved in the increased hepatic uptake of circulating triglycerides [21], especially around birth and throughout the suckling period [22]. The present study also confirms an increased fatty acid beta-oxidation in the offspring of dams fed an SPI diet, which have significantly higher mRNA levels of *Cpt1b* and *Hadh* than the offspring of dams fed a CAS diet. Fetal liver has been shown to contain a mixture of adult liver- and muscle-type of CPT1 [23]. Thus, an increased fatty acid oxidation may occur to compensate for an increased uptake of free fatty acids into the liver, thereby resulting in similar hepatic triglyceride levels between the CAS and SPI groups. Additional downregulation of mRNA levels of a lipogenic gene, *Fasn*, may contribute to the hypotriglyceridemic effect of the maternal SPI diet compared with the maternal GEN diet. The mRNA levels of other lipogenic genes, *Thrsp* and *Scd1*, tended to be lower in the SPI group compared with the CAS and GEN groups. 

We found that the hepatic expression of some selected genes important in the cholesterol metabolism was also altered by maternal diet (Figure 3b). There was a distinct regulation of gene expression in the offspring by maternal SPI and GEN diet. In the SPI group, mRNA levels of *Cyp3a1*, a gene that plays an important role in the hydroxylation and excretion of bile acid in the liver [24], were significantly higher compared with those in the CAS and GEN groups. Maternal consumption of a GEN diet, but not an SPI diet, significantly increased the mRNA levels of *Ldlr*, suggesting that increased lipoprotein uptake may be involved in the hypocholesterolemic effect observed in the GEN group. Furthermore, we observed that significantly higher *Abca1* mRNA levels in the GEN group compared with the SPI group suggested the increased reverse cholesterol transport, as indicated by the ratio of HDL cholesterol to total cholesterol. In contrast, *Lcat* mRNA levels were significantly lower in the SPI group compared with the CAS group (*p* = 0.020, by *t*-test), which suggested an inhibition of pre-beta HDL (nascent HDL) maturation to alpha-HDL (mature HDL) in the SPI group. *Apoa2* mRNA levels were significantly increased by maternal SPI consumption. Therefore, the maturation of nascent HDL rather than the biosynthesis of nascent HDL may be suppressed in the SPI group compared with the other two groups. For comparison, microarray data are shown in Supplementary Figure S2.

### 3.6. Effects of Maternal Diet on the Activation of Peroxisome Proliferator-Activated Receptor Alpha (PPARα) Signaling Pathway in the Liver of Offspring

**Figure 4:**
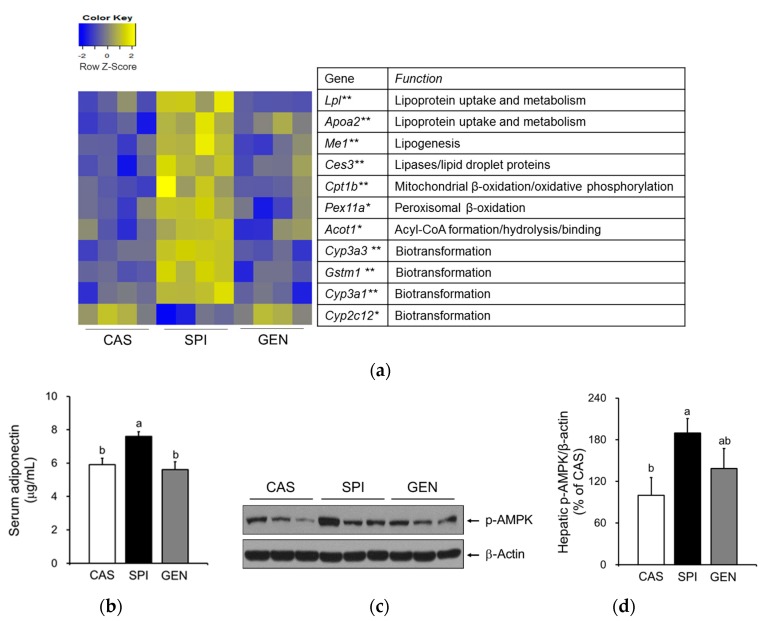
Effects of maternal diet on hepatic expressions of peroxisome proliferator-activated receptor alpha (PPARα)-target genes of male offspring. (**a**) Visualization of gene expression levels as a heat map. Data were Z-score normalized. Each cell represents the individual differentially expressed genes in each offspring liver sample (*n* = 4). Yellow color represents the up-regulated gene expression and blue color represents the down-regulated gene expression. * *p* < 0.05 and ** *p* < 0.01 for maternal diet effect among groups; (**b**) Serum adiponectin (*n* = 9) was measured by ELISA; (**c**,**d**) Hepatic phosphorylated AMP-activated protein kinase (p-AMPK) protein levels were determined by immunoblotting (*n* = 4–5). Data are means ± standard error of the mean. Means with different superscripts are significantly different at *p* < 0.05.

Maternal diet did not alter mRNA levels of PPARα, a gene encoding a key transcription factor that regulates many aspects of lipid metabolism such as fatty acid oxidation, uptake, and transport, in the liver of offspring. However, we observed increased expressions of PPARα target genes, including *Me1*, *Cpt1b*, *Lpl*, *Cyp3a1,* and *Apoa2* in the livers of the SPI group (Figure 3a,b). To confirm the altered PPARα signaling pathway in the SPI group, we identified 11 PPARα target genes among 119 differentially expressed genes in response to maternal diet (*p* < 0.05) with a fold change equal to or greater than 1.5 between any two groups based on a previous study [25]. Most genes, except *Cyp2c12*, were significantly induced in response to maternal SPI diet (Figure 4a). A previous study reported that *Cyp2c12* was downregulated by peroxisome proliferators [26]. Furthermore, serum levels of adiponectin, which is associated with the activation of PPARα pathways [27], were significantly increased in the SPI group compared with the CAS group (Figure 4b). Furthermore, the activation of AMPK, another downstream pathway of adiponectin in the liver, was significantly induced in the SPI group compared with the CAS group (Figure 4c,d).

### 3.7. Effects of Maternal Biochemical Parameters on the Regulation of Lipid Metabolism of Offspring

**Figure 5:**
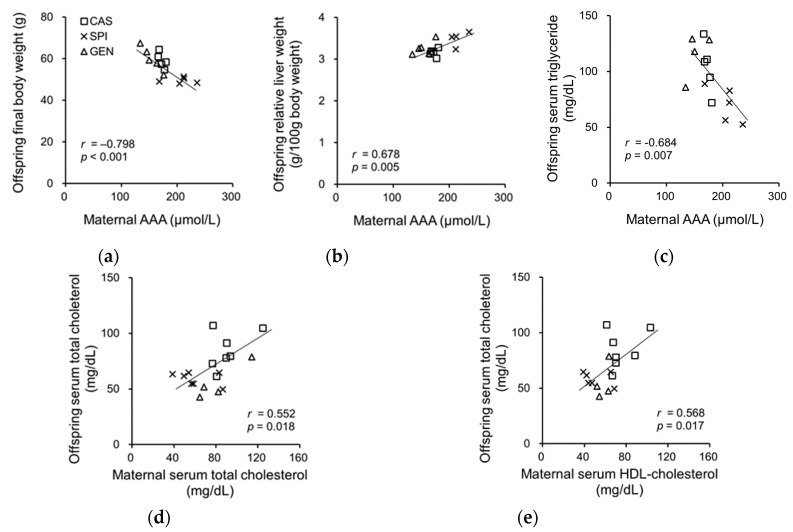
Correlation between (**a**) serum AAA levels in dams and body weight; (**b**) serum AAA levels in dams and relative liver weight of offspring; (**c**) serum AAA levels in dams and serum triglyceride levels in offspring; (**d**) serum total cholesterol levels in dams and serum total cholesterol levels in offspring; (**e**) serum high-density lipoprotein (HDL) cholesterol levels in dams and serum total cholesterol levels in offspring AAA, aromatic amino acid. Pearson correlation coefficient, *r* and *p*-value are indicated.

To investigate the related maternal factors determining the lipid metabolism of offspring, we investigated the association between the serum biochemical parameters of dams and the anthropometrical and serum biochemical parameters of offspring. Previously, we have reported that the SPI group had significantly lower body weight and relative adipose tissue weight and significantly higher relative liver weight compared with the CAS group [15]. Offspring body weight was negatively associated with the serum AAA levels in dams, whereas the relative liver weight was positively associated with the serum AAA levels of dams (Figure 5a,b). We also observed a significant correlation between the serum triglyceride levels of offspring and the serum AAA levels of dams (Figure 5c), but not with the serum triglyceride levels of dams (*p* = 0.177). The serum cholesterol levels of offspring were significantly associated with serum cholesterol (Figure 5d) and the HDL cholesterol levels of dams (Figure 5e). There were no significant associations between serum insulin, adiponectin, and T3 levels in dams, and the anthropometrical parameters and serum lipid levels of offspring (data not shown).

**Figure 6:**
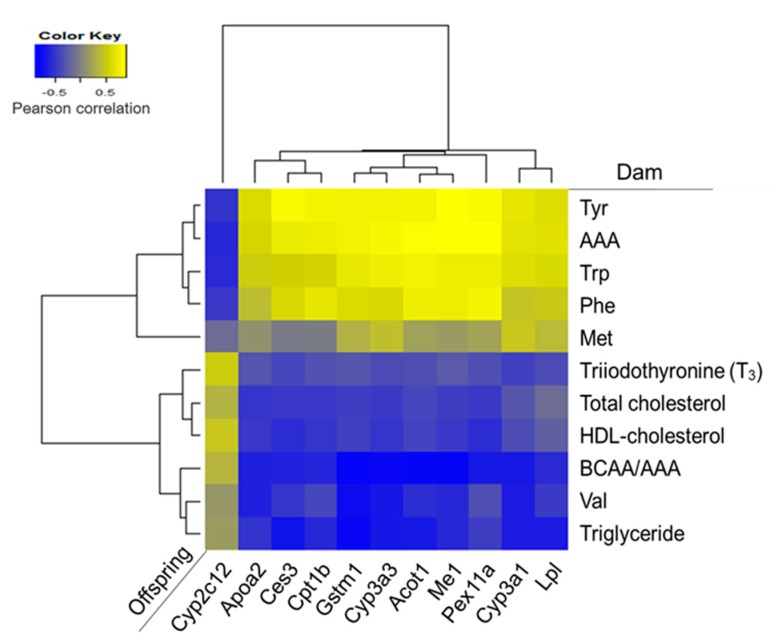
Heat map of Pearson correlation coefficients between serum parameters in dams and hepatic expressions of PPARα-target genes in offspring. Yellow color represents positive correlation and blue color represents negative correlation.

Pearson correlation coefficients were calculated between the serum biochemical variables that were significantly different among the dam groups, and the mRNA levels of PPARα-target genes in offspring (Figure 6). Most mRNA levels of PPARα-target genes showed strong positive correlations with serum tyrosine and AAA levels in dams.

## 4. Discussion

Several studies have suggested that the consumption of isoflavone with soy protein has different biological effects than the consumption of isoflavone without soy protein [28]. Previously, we have reported that maternal intake of low-isoflavone SPI significantly reduced body weights and induced liver growth retardation in offspring, with substantial effects on hepatic gene expression [15]. Therefore, we investigated whether dietary protein source and isoflavone contents in the maternal diet would regulate the lipid metabolism in their offspring on PND 21. Lipid metabolism-related genes were shown to be significantly upregulated in mouse liver during PND 7–PND 21 [29]. 

In the present study, serum triglyceride, total cholesterol, and HDL cholesterol levels were significantly lower in the SPI group compared with the CAS group. In contrast, only total serum cholesterol levels were significantly lower in the GEN group compared with the CAS group, resulting in a significant increase in the ratio of HDL cholesterol to total cholesterol. No significant differences in hepatic triglyceride and cholesterol levels were observed among the groups. Maternal SPI consumption attenuated serum lipid levels in offspring by reducing hepatic lipogenesis, increasing fatty acid oxidation, and enhancing bile acid catabolism based on the hepatic mRNA levels of related genes.

Although both serum triglyceride and total cholesterol levels were decreased in the SPI group compared to the CAS group, it seems that different mechanisms are involved in maternal effects on lipid metabolism of offspring. Triglycerides in milk are about 100-fold higher than those in the serum of dams and their offspring [30], suggesting that lower serum triglyceride levels in the SPI group were not due to lower triglyceride levels in the milk of their dams. Meanwhile, dams at 21 days of suckling fed a soy protein diet showed lower cholesterol levels in serum, liver, and milk compared with dams fed a casein diet [31]. Similarly, maternal flaxseed diet during lactation decreased cholesterol levels in milk and serum of offspring [32]. Therefore, cholesterol contents in milk could possibly affect serum cholesterol levels. 

In consideration of the regulatory role of PPARα on lipid metabolism in adults, we examined whether PPARα activation in offspring is altered by maternal diet. Our results clearly showed increased expressions of target genes of PPARα in the SPI group, which may be responsible for the hypolipidemia observed in the SPI group. PPARα has been shown to regulate the transcription of genes involved in glucose and lipid metabolism, liver inflammation, and hepatocyte proliferation (especially in rodents) [25]. Besides natural ligands, including fatty acids and their metabolites, various xenobiotics are shown to activate PPARs [33]. Indeed, we observed increases in the mRNA levels of genes involved in xenobiotics and drug metabolism. The altered levels of xenobiotics were possibly due to the retarded liver development, and may activate PPARα in the SPI group [15]. Interestingly, the mRNA levels of PPARα-target genes, including *Me1*, *Cpt1b*, *Lpl*, *Cyp3a1,* and *Apoa2* were significantly associated with the relative liver weight of offspring (data not shown). Similarly, the induction of dyslipidemia in offspring by a maternal low-protein diet (9% vs. 18%) during pregnancy involves an altered epigenetic regulation of specific transcription factors, including PPARα, in the liver of offspring [34]. Indeed, a recent study reported the increased gene expressions of the PPARα pathway (*Cyp7a1*, *Aqp7*, *Lpl* and *Cpt1b*) by maternal low-protein diet (8% protein) throughout pregnancy and lactation in the liver of mice offspring [35]. Furthermore, the maternal consumption of oxidized fat during pregnancy upregulates expression of PPARα-target genes with lowered triglyceride concentrations in both dam and fetal livers [36]. 

Although structure–activity relationship studies showed a direct binding of isoflavones to PPARs [37], we did not observe the PPARα activation in the livers of GEN diet-fed dams (data not shown) and their offspring. Instead, we observed the significant increase in *Ldlr* mRNA levels in the GEN group. Consistently, a previous study showed a higher expression of the *Ldlr* gene in male Zucker rats fed a high-isoflavone diet compared with casein-fed rats, but not in rats fed a protein diet with similar amino acid profiles (low methionine/glycine and low lysine/arginine) with SPI for six weeks [38]. 

A previous study proposed that non-isoflavone phytochemicals and their metabolites, or the altered protein/peptide composition during isoflavone-removal processing, may be responsible for the effect of SPI on PPARα activation [39]. A recent cell culture study reported that the soybean-derived dipeptide Trp-Glu is a key hypolipidemic compound *via* PPARα activation [40]. Here, we did not observe a higher activation of PPARα in dams fed an SPI compared with dams fed a CAS diet (data not shown), which suggested that hormonal or metabolic changes induced in dams may be responsible for PPARα activation in the SPI group. 

Maternal consumption of a SPI diet significantly decreased serum BCAA/AAA in dams compared with a CAS diet (1.5 vs. 2.3). In normal animals, the BCAA/AAA ratio in plasma is about 3:1 [41]. Previous studies reported decreases in maternal BCAA plasma concentrations in intrauterine growth restriction (IUGR) models with dams fed a low-protein diet, and in *Ldlr*^−/−^ dams fed a Western diet [42,43]. Similarly, IUGR induced by calorie restriction decreased plasma concentrations of BCAAs [44]. In the same study, BCAA supplementation effectively increased plasma BCAA levels and improved the physiological functions of uterus and placenta, resulting in increased litter size and embryo weight [44]. These results suggest that the altered amino acid profile, especially lowered BCAA concentrations, may be responsible for some of the phenotypes observed in the offspring of IUGR models. We consistently observed a significant negative correlation between the serum AAA levels in dams and the body weight of offspring at PND 21. Moreover, maternal AAA concentration was significantly correlated with serum triglyceride levels and PPARα-target gene expressions in offspring. Further research is warranted to investigate the underlying mechanism for the association between maternal AAA levels and PPARα activation in offspring. 

## 5. Conclusions

Maternal consumption of low-isoflavone SPI and genistein differently regulated serum lipid parameters and hepatic gene expressions involved in lipid metabolism in offspring. Interestingly, more pronounced effects on lipid metabolism were observed in the offspring of SPI diet-fed dams, suggesting the important role of maternal SPI consumption regardless of isoflavone levels in the lipid metabolism regulation of offspring.

## Figures and Tables

**Table 1:** Composition of experimental diet.

Composition (g/kg)	Diet		
	CAS	SPI	GEN
Cornstarch	397.5	397.5	397.5
Casein ^1^	200	-	200
Soy protein isolate ^2^	-	200	-
Dextrinized cornstarch	132	132	132
Sucrose	100	100	99.75
Corn oil	70	70	70
Fiber	50	50	50
Mineral mix ^3^	35	35	35
Vitamin mix ^4^	10	10	10
L-Cystine	3	3	3
Choline bitartrate	2.5	2.5	2.5
*t*-Butylhydroquinone	0.014	0.014	0.014
Genistein ^5^	-	-	0.25

^1^ Protevit-S (Lactoprot Deutschland GmbH, Kaltenkirchen, Germany); ^2^ PRO-FAM^®^ 974 (ADM, Chicago, IL, USA); ^3^ AIN-93G-MX (Dyets Inc., Bethlehem, PA, USA); ^4^ AIN-93-VX (Dyets Inc., USA); ^5^ Genistein (Chromadex Inc., Irvine, CA, USA). CAS, casein; SPI, low-isoflavone soy protein isolate; GEN, casein plus genistein.

**Table 2:** Effects of diets on the serum and hepatic biochemical parameters of dams.

	Group		
	CAS-D	SPI-D	GEN-D
Body weight at sacrifice (g)	299.5 ± 12.6	299.9 ± 3.4	304.0 ± 11.2
Serum			
Glucose (mg/dL)	94.5 ± 6.6	99.3 ± 10.1	104.2 ± 13.3
Triglyceride (mg/dL)	81.8 ± 10.4 ^a^	48.7 ± 7.3 ^b^	80.4 ± 15.5 ^a^
Total cholesterol (mg/dL)	92.0 ± 5.7 ^a^	59.4 ± 6.0 ^b^	77.0 ± 8.2 ^a,b^
HDL cholesterol (mg/dL)	74.8 ± 4.9 ^a^	49.0 ± 4.8 ^b^	56.9 ± 4.1 ^b^
Free fatty acids (μmol/L)	722.1 ± 88.5	617.1 ± 32.1	694.9 ± 75.4
Homocysteine (μmol/L)	6.9 ± 0.4	7.6 ± 0.5	6.9 ± 0.4
Insulin (ng/mL)	0.5 ± 0.1	0.3 ± 0.1	0.9 ± 0.4
Adiponectin (ng/mL)	6.7 ± 0.4	8.1 ± 0.8	6.3 ± 0.7
T3 (ng/mL)	1.7 ± 0.1 ^a^	1.4 ± 0.1 ^a,b^	1.2 ± 0.2 ^b^
Liver			
Triglyceride (μg/mg protein)	81.0 ± 2.7 ^a^	63.5 ± 3.9 ^b^	87.3 ± 5.2 ^a^
Cholesterol (μg/mg protein)	5.8 ± 1.3 ^a^	2.5 ± 0.5 ^b^	5.4 ± 0.6 ^a^

Data are means ± standard error of the mean (*n* = 6–9 for anthropometric and hepatic parameters, *n* = 5–8 for serum parameters). Means in the same row with different superscript are significantly different at *p* < 0.05. CAS-D, dams fed a casein diet; SPI-D, dams fed a low-isoflavone soy protein isolate diet; GEN-D, dams fed a casein plus genistein diet; HDL, high-density lipoprotein.

**Table 3:** Effects of diets on serum amino acid profiles in dams.

Amino Acid (μmol/L)	Group		
	CAS-D	SPI-D	GEN-D
Alanine	489.2 ± 44.7	464.5 ± 33.7	490.1 ± 13.8
Arginine	170.6 ± 5.0	191.3 ± 13.3	141.0 ± 23.3
Asparagine	76.8 ± 3.6	74.7 ± 6.6	72.2 ± 1.5
Aspartate	47.8 ± 3.0	48.8 ± 9.4	40.3 ± 4.3
Glutamate	186.9 ± 9.2	198.2 ± 24.0	203.6 ± 24.0
Glutamine	610.5 ± 42.9	550.7 ± 34.1	513.7 ± 30.8
Glycine	234.7 ± 16.0	255.6 ± 25.8	225.1 ± 20.5
Histidine	56.0 ± 1.2	52.1 ± 2.8	50.3 ± 1.3
Isoleucine	95.8 ± 6.9	80.1 ± 3.4	85.3 ± 8.1
Leucine	145.5 ± 9.5	117.6 ± 5.7	129.2 ± 12.6
Lysine	477.4 ± 25.0	477.7 ± 62.0	431.1 ± 47.5
Methionine	52.9 ± 1.2 ^a^	52.9 ± 1.6 ^a^	40.2 ± 6.2 ^b^
Phenylalanine	61.9 ± 1.5 ^a,b^	63.2 ± 3.4 ^a^	54.1 ± 2.6 ^b^
Proline	172.4 ± 6.5	148.9 ± 9.8	169.5 ± 11.2
Serine	416.9 ± 18.0	401.3 ± 24.8	373.7 ± 12.7
Threonine	662.0 ± 97.0	426.3 ± 37.3	608.9 ± 100.9
Tryptophan	62.9 ± 1.4 ^b^	78.8 ± 6.6 ^a^	53.5 ± 3.7 ^b^
Tyrosine	47.8 ± 1.9 ^b^	64.6 ± 5.8 ^a^	46.2 ± 3.2 ^b^
Valine	152.4 ± 8.6 ^a^	119.4 ± 3.5 ^b^	137.7 ± 10.4 ^a,b^
BCAA	393.7 ± 24.6	317.1 ± 12.4	352.1 ± 30.7
AAA	172.5 ± 2.7 ^b^	206.6 ± 11.0 ^a^	153.9 ± 7.3 ^b^
BCAA/AAA	2.3 ± 0.1 ^a^	1.5 ± 0.1 ^b^	2.3 ± 0.2 ^a^

Data are means ± SEM (*n* = 5). Means in the same row with different superscript are significantly different at *p* < 0.05. BCAA, branched-chain amino acids (isoleucine, leucine and valine); AAA, aromatic amino acids (phenylalanine, tryptophan and tyrosine).

## Supplementary Materials

The following are available online at www.mdpi.com/2072-6643/9/9/1039/s1. Table S1: List of primers used for real-time PCR (TaqMan); Table S2: Primer sequences for real-time PCR (SYBR green); Figure S1: Effects of diet on body weight of dams during pre-pregnancy, pregnancy and lactation. Data are means ± standard effort of the mean (*n* = 6–9). Means with different superscripts are significantly different at *p* < 0.05. CAS-D, dams fed a casein diet; SPI-D, dams fed a low-isoflavone soy protein isolate diet; GEN-D, dams fed a casein plus genistein diet; Figure S2: Effects of maternal diet on hepatic gene expression related to lipid metabolism of male offspring. Each column of heat map represents the mean of the individual differentially expressed genes in maternal diet group (*n* = 4). Data were Z-score normalized. Yellow color represents the up-regulated gene expression compared to the mean, whereas blue color represents the down-regulated gene expression below the mean.
