# Supplementary material for: Different Effects of Maternal Low-Isoflavone Soy Protein and Genistein Consumption on Hepatic Lipid Metabolism of 21-Day-Old Male Rat Offspring
Source: Nutrients. 2017 Sep 20;9(9):1039. doi: 10.3390/nu9091039 (PMC5622799; doi:10.3390/nu9091039)
Supplement: Supplementary file 1 [file nutrients-09-01039-s001.pdf]

Supplementary Table S1. List of primers used for real-time PCR (TaqMan).

Gene	Product number	Gene	Product number
Abca1	Rn00710172_m1	*Hadh*	Rn00589352_m1
Abcg5	Rn00587092_m1	*Hmgcr*	Rn00565598_m1
Acot12	Rn00590651_m1	*Lcat*	Rn00500505_m1
Apoa2	Rn00565403_m1	*Ldlr*	Rn00598442_m1
Cd36	Rn00580728_m1	*Lpl*	Rn00561482_m1
Cpt1a	Rn00580702_m1	*Me1*	Rn00561502_m1
Cpt1b	Rn00566242_m1	*Mttp*	Rn01522970_m1
Cyp3a1	Rn03062228_m1	*Ppara*	Rn00566193_m1
Cyp7a1	Rn00564065_m1	*Scd1*	Rn00594894_g1
Dgat1	Rn00584870_m1	*Srb1*	Rn00580588_m1
Fasn	Rn00569117_m1	*Thrsp*	Rn01511034_m1
Actb	Rn00667869_m1		

Abca1, ATP-binding cassette, subfamily A, member 1; *Abcg5*, ATP-binding cassette, subfamily G (WHITE), member 5; *Acot12*, acyl-CoA thioesterase 12; *Apoa2*, Apo-lipoprotein A2; *Cd36*, Cluster of differentiation 36; *Cpt1a*, Carnitine palmitoyltransferase 1α; *Cpt1b*, Carnitine palmitoyltransferase 1b; *Cyp3a1*, Cytochrome P450, family 3, subfamily a, polypeptide 1; *Cyp7a1*, Cholesterol 7α hydroxylase; *Dgat1*, Diacylglycerol O-acyltransferase homolog 1; *Fasn*, Fatty acid synthase; *Hadh*, hydroxyacyl-Coenzyme A dehydrogenase; *Hmgcr*, 3-Hydroxy-3-methylglutaryl-Coenzyme A reductase; *Lcat*, Lecithin cholesterol acyltransferase; *Ldlr*, Low density lipoprotein receptor; *Lpl*, Lipoprotein lipase; *Me1*, Malic enzyme 1; *Mttp*, Microsomal triacylglycerol transfer protein; *Ppara*, peroxisome proliferator activated receptor alpha; *Scd1*, Stearoyl-CoA desaturase 1; *Srb1*, Scavenger receptor class B, member 1; *Thrsp*, Thyroid hormone responsive protein; *Actb*, actin, beta.

Supplementary Table S2. Primer sequences for real-time PCR (SYBR green).

Gene	Forward (5′- 3′)	Reverse (5′- 3′)
Apoa1	GGCAGAGACTATGTGTCCCAGTTT	TTGAACCCAGAGTGTCCCAGTT
Dgat2	ATGCCTGTGCTTCGGGAGTA	ACCTCCCACCACGATGACAA
Lxra	GAACAGATCCGCTTGAAGAA	ATGGCCAGCTCAGTAAAGTG
Actb	CACACTGTGCCCATCTATGA	CCGATAGTGATGACCTGACC

Apoa1, Apo-lipoprotein A1; *Dgat2*, Diacylglycerol O-acyltransferase homolog 2; *Lxra*, Liver X receptor alpha; *Actb*, actin, beta.

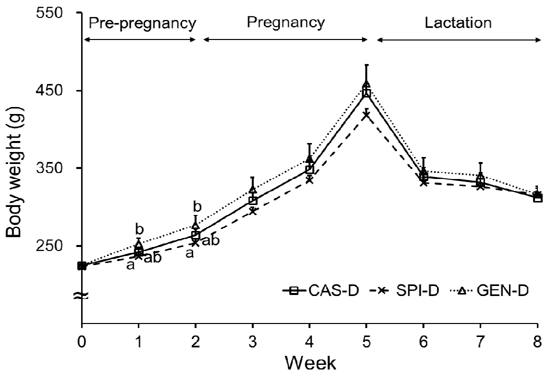

Supplementary Figure S1. Effects of diets on body weight of dams during pre-pregnancy, pregnancy, and lactation. Data are means ± standard error of the mean ($n = 6–9$). Means with different superscripts are significantly different at $p < 0.05$. CAS-D, dams fed a casein diet; SPI-D, dams fed a low-isoflavone soy protein isolate diet; GEN-D, dams fed a casein plus genistein diet.

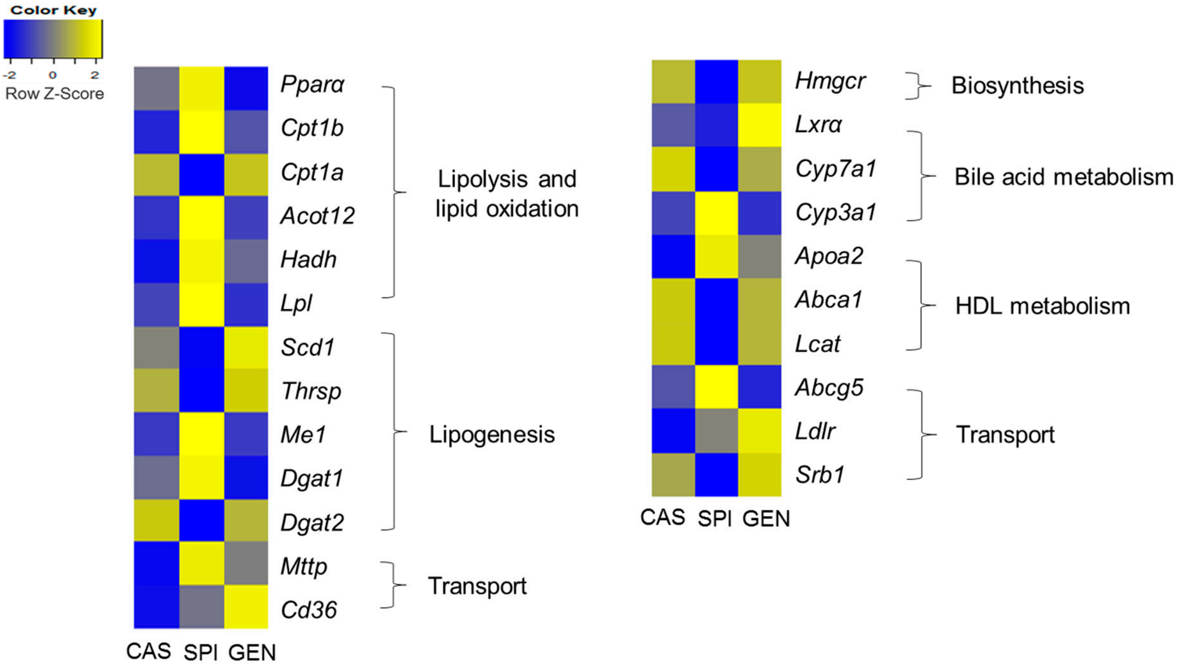

Supplementary Figure S2. Effects of maternal diet on hepatic gene expression related to lipid metabolism of male offspring. Each column of heat map represents the mean of the individual differentially expressed genes in maternal diet group ($n = 4$). Data were Z-score normalized. Yellow color represents the upregulated gene expression compared with the mean, whereas blue color represents the downregulated gene expression below the mean.
